# The role of reactive oxygen species in severe acute respiratory syndrome coronavirus 2 (SARS-COV-2) infection-induced cell death

**DOI:** 10.1186/s11658-024-00659-6

**Published:** 2024-11-08

**Authors:** Jiufeng Xie, Cui Yuan, Sen Yang, Zhenling Ma, Wenqing Li, Lin Mao, Pengtao Jiao, Wei Liu

**Affiliations:** 1 College of Life Sciences, Henan Agricultural University, Zhengzhou, 450002 China; 2 CAS Key Laboratory of Pathogenic Microbiology and Immunology, Institute of Microbiology, Chinese Academy of Sciences, Beijing, 100101 China

**Keywords:** SARS-CoV-2, COVID-19, Reactive oxygen species, Cell death, Antiviral therapy

## Abstract

Coronavirus disease 2019 (COVID-19) represents the novel respiratory infectious disorder caused by severe acute respiratory syndrome coronavirus 2 (SARS-CoV-2) and is characterized by rapid spread throughout the world. Reactive oxygen species (ROS) account for cellular metabolic by-products, and excessive ROS accumulation can induce oxidative stress due to insufficient endogenous antioxidant ability. In the case of oxidative stress, ROS production exceeds the cellular antioxidant capacity, thus leading to cell death. SARS-CoV-2 can activate different cell death pathways in the context of infection in host cells, such as neutrophil extracellular trap (NET)osis, ferroptosis, apoptosis, pyroptosis, necroptosis and autophagy, which are closely related to ROS signalling and control. In this review, we comprehensively elucidated the relationship between ROS generation and the death of host cells after SARS-CoV-2 infection, which leads to the development of COVID-19, aiming to provide a reasonable basis for the existing interventions and further development of novel therapies against SARS-CoV-2.

**Graphical Abstract:**

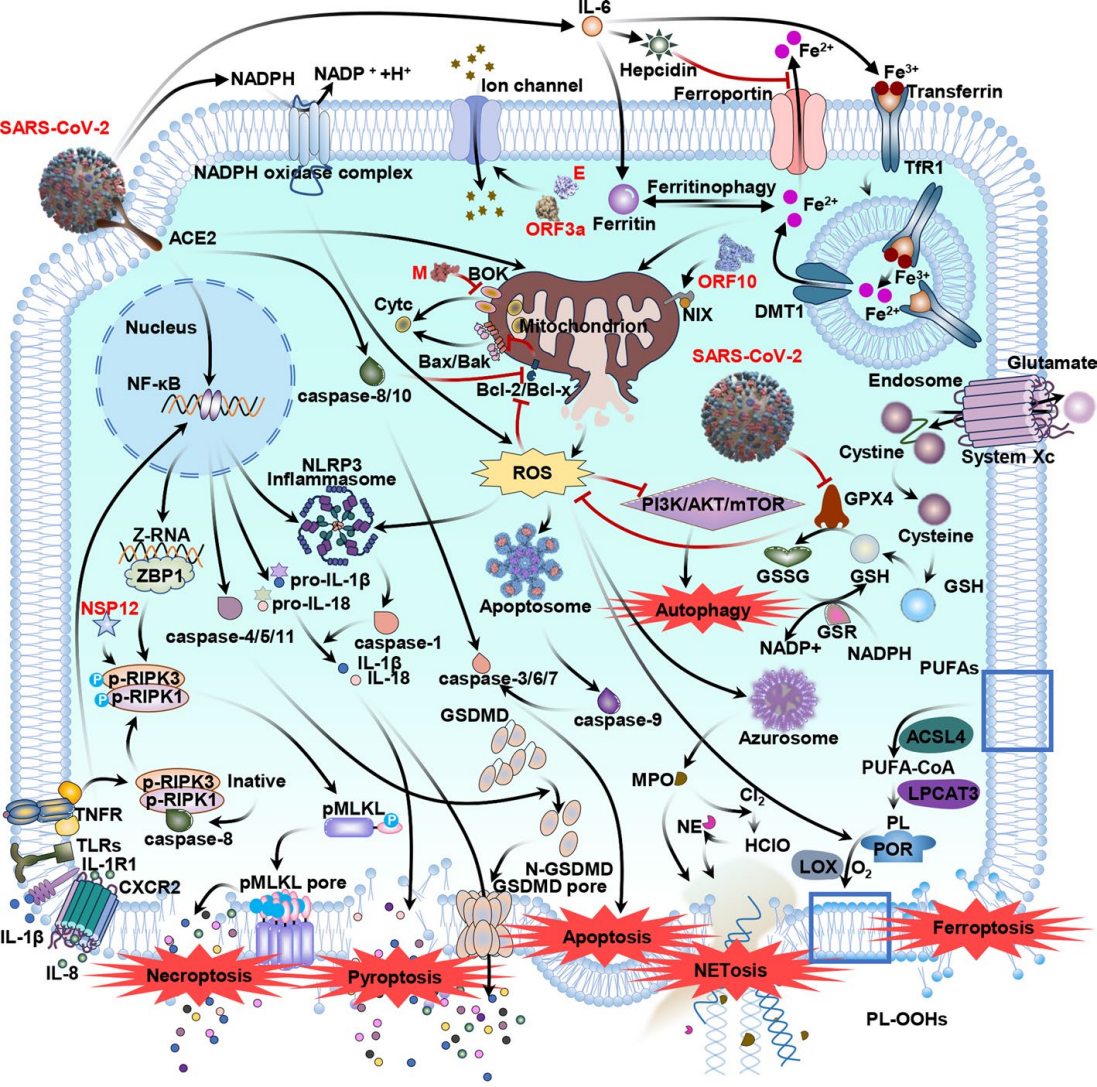

## Introduction

The novel coronavirus disease 2019 (COVID-19) was first reported in January 2020. It was caused by the highly contagious severe acute respiratory syndrome coronavirus-2 (SARS-CoV-2), which spread throughout the world and became a severe public health issue. As of 14 September 2024, the cumulative number of reported cases by the World Health Organization (WHO) stands at 776,007,137, with a total of 7,059,612 deaths (for continuously updated information, refer to: https://covid19.who.int). SARS-CoV-2 can bind to angiotensin-converting enzyme-2 (ACE2) or CD147 receptors on the cell membrane to infect host cells with the help of protease Furin and transmembrane serine protease 2 (TMPRSS2). RNA replication and protein expression occur in SARS-CoV-2 after it enters host cells. Once entering into the host cell, SARS-CoV-2 replicates its RNA and expresses proteins, assembles into complete viral particles and eventually releases virions, concurrently leading to death of host cell and release of cellular content [1]. SARS-CoV-2 shows a high infection rate in the lungs and the respiratory tract, inducing the novel coronavirus pneumonia. COVID-19 exhibits different types of clinical manifestations, ranging from mild to severe, including asymptomatic infection, pulmonary or extrapulmonary symptoms (e.g., fever, cough, fatigue, nausea, diarrhea and loss of smell and taste), acute respiratory distress syndrome (ARDS), acute kidney injury (AKI), multiple organ failure and even death [2].

Oxidative stress represents the non-specific pathological state that reflects the imbalance between ROS generation and antioxidant capacity. ROS are generally produced as oxygen metabolic by-products. They strongly affect the modulation of cell survival [3], differentiation [4], cell death [5], cell signalling [6] and generation of inflammatory factors [7]. ROS have multiple effects on the immune system; they are also closely related to different immune response aspects, including the activation and interaction of immune cells, host defense and immune suppression [8]. Several studies have shown that ROS accumulation can promote senescence and several processes of cell death [9]. Oxidative stress has a crucial effect on virus infections like SARS-CoV-2 infections. Therefore, understanding the role of ROS in modulating host cell death following SARS-CoV-2 infection is extremely important.

SARS-CoV-2 infection can induce immune hyperactivation, causing a cytokine storm, which plays a key role in inducing pulmonary injury, critical prognosis and multiple organ failure [10]. In a viral infection, the above systems are dysregulated, which facilitates disease pathogenesis. Cell death mechanisms help maintain an optimum environment to execute appropriate cellular activities [11]. The process of cell death is regulated by various signaling pathways that originate from either the external environment of the cell (extrinsic inducers) or from within the cell itself (intrinsic inducers). Extrinsic inducers encompass factors such as heat, radiation, toxins, nitric oxide and hormones [12]. These inducers must either penetrate the cell or interact with specific receptors located on the cell membrane to initiate the corresponding signal transduction pathways. This interaction subsequently induces the cell to undergo programmed cell death. Several intrinsic factors, including the accumulation of misfolded proteins, intracellular ATP depletion, nutrient deprivation, lipid peroxidation and oxidative stress, can lead to cell death. Thus, during viral infection, any external or internal disturbance in the signal transduction pathways within a cell may lead the cell to undergo cell death [13]. SARS-CoV-2 infection may activate different cell death pathways in host cells, including apoptosis, ferroptosis, pyroptosis, NETosis, necroptosis and autophagy [14]. Upon SARS-CoV-2 infection, these cell death pathways have certain similar characteristics, including their dependence on ROS signalling and control. ROS produced by cells with low antioxidant ability may regulate cell survival or death, as well as the associated cell death mechanism.

ROS influence immune response (mostly associated with innate immunity) to viruses in different ways. Thus, many researchers have investigated how ROS contribute to cell death in severe COVID-19. The relationship among SARS-CoV-2, ROS and cell death remains unclear. In this article, we mainly discussed the effect of ROS on various modes of cell death resulting from SARS-CoV-2 infection and analysed the anti-COVID-19 therapeutic targets on molecules related to the cell death pathway.

## ROS generation following SARS-COV-2 infection

ROS are oxygen-containing molecules with high reactivity and include hydrogen peroxide, superoxide anion, lipid peroxides, hydroxyl radical and protein peroxides, as well as, peroxides generated in nucleic acids [15]. Viral infection can promote ROS production, which disrupts redox homeostasis and elicits inflammation, oxidative stress and biological responses tightly associated with disease progression. During infection, ROS generation is probably promoted through the response of cytokines to pathogens or viral components. For example, the expression of non-structural protein 1 (nsp1) of Middle East respiratory syndrome coronavirus (MERS-CoV) increases intracellular ROS content, thus activating AMPK while inhibiting the mTOR pathway and inducing autophagy [16]. The PB1-F2 protein of the H7N9 influenza A virus (IAV) is a major virulence factor that can induce mitochondrial ROS production and trigger inflammasome activation and interleukin-1β (IL-1β) secretion by downregulating superoxide dismutase 1 (SOD1) [17].

Respiratory viruses generally promote ROS production in host cells. SARS-CoV-2 increases oxidative stress in host cells; they can aggravate oxidative stress in host cells by activating pro-oxidant enzymes and inhibiting antioxidant enzymes, enhancing ROS and neutrophil extracellular trap (NET) burst and increasing hypoxia-mediated ROS generation. Several studies have suggested that ROS strongly influences different modes of cell death, particularly in the early immune response stage. The ability of ROS to determine different cell death modes has received much attention. Many researchers have also shown that ROS strongly influences SARS-COV-2 infection-mediated cell death.

## Effect of ROS on different types of cell death induced by SARS-COV-2 infection

Cell death-induced cytopathy resulting from viruses that invade host cells occurs commonly following a viral infection. Under certain circumstances, cell death may suppress the replication of viruses. It mostly promotes virus dissemination and affects cellular physiology, causing injury to tissues and organs [18]. Coronavirus replication in cells can be controlled by numerous host factors (including ROS), which can induce substantial cellular physiological and structural changes. In the process of infection, SARS-CoV-2 may promote different cell death pathways [19], such as ferroptosis, NETosis, apoptosis, autophagy and pyroptosis of the host cells.

### NETosis

NETosis is the typical ROS-dependent programmed cell death (PCD) that occurs in neutrophils. The typical features of NETosis include the extrusion of DNA, antimicrobial proteins and histones within the web-like structure called neutrophil NETs. NETosis can occur due to various proinflammatory factors (such as IL-8 and IL-1β) or microbial stimuli [20]. An increase in ROS production is an important intracellular process causing NETosis. ROS can activate proteases such as GSDMD, neutrophil elastase (NE) and protein arginine deiminase 4 (PAD4), which are responsible for catalysing cell rupture, nuclear disintegration and chromatin decondensation [20]. Irrespective of the critical role of NETs in suppressing pathogen invasion, excess NET generation may lead to various adverse effects, including tissue injury and autoimmune inflammation. The activation of circulating NETs can also promote thrombosis and hypercoagulability [21].

NETosis and the release of NETs can increase by infiltrating and circulating neutrophils among patients with COVID-19 [22]. Some studies have shown that SARS-CoV-2 infection can induce NETosis and the release of NETs in normal neutrophils. SARS-CoV-2 promotes an oxidative burst in neutrophils and inhibits antioxidant response, thus deteriorating immune response in the context of COVID-19, such as NETs and NETosis. Arcanjo et al. found that SARS-CoV-2 can induce NETosis in human neutrophils by promoting ROS generation [23]. The self-sustained IL-8 autocrine production loop, which is also an important and inherent driving factor for NETosis, is present in peripheral blood and pulmonary neutrophils. It can promote NET generation and indicate the severity of COVID-19. NET generation is the predicting factor for the severity of COVID-19 and clinical prognosis. Immunothrombosis induction is an important mechanism underlying NETosis and NET-associated disorders [24]. NETs and NETosis were also found to be the mediating factors for additional pathophysiological alterations, including neurological abnormalities, immune dysfunction and post-COVID-19 diseases.

**Fig. 1:**
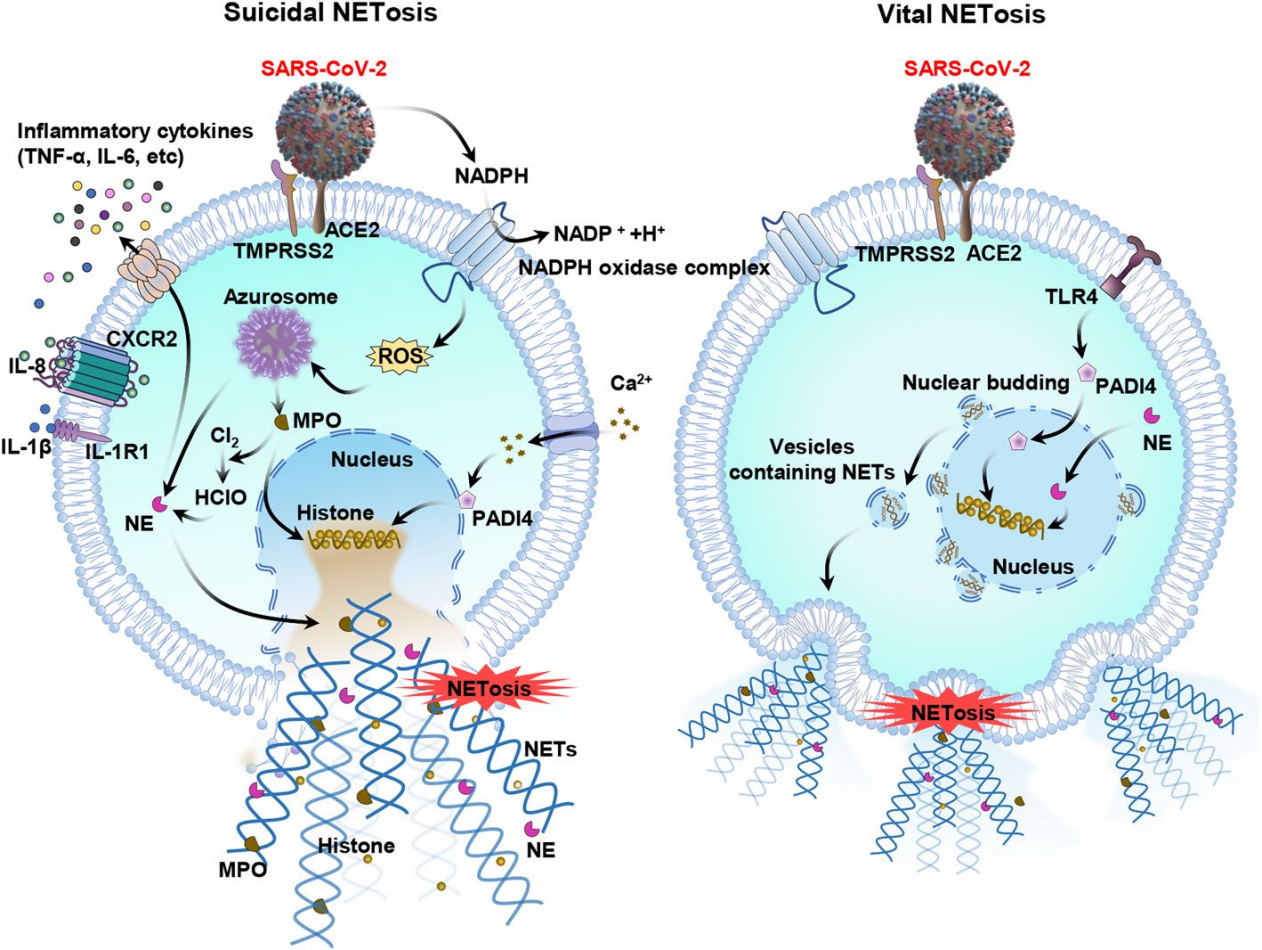
Effects of ROS on the NETosis pathway in SARS-CoV-2 infection. Suicidal NETosis, in resting neutrophils, NE and MPO were preserved in azurophilic granules. In the case of SARS-CoV-2 infection and ROS generation, NE escapes from the granules and translocates to the nucleus. In the nucleus, NE cleaves histones and accelerates chromatin decondensation. MPO combines with chromatin during the late stage and enhances decondensation. NE and MPO together promotes chromatin decondensation, causing cell rupture and the release of NET. TLR4 is required in vital NETosis, SARS-CoV-2-mediated NET formation, in a NOX-produced ROS-independent manner. The activated NE and PAD4 cause chromatin decondensation, thereby forming NET-filled vesicles. These vesicles are expelled from the nucleus and enter the extracellular space without disrupting the cell membrane. The integrity of the cell membrane is maintained during vital NETosis and cell functions are maintained. Activation effects are indicated by black arrows

Suicidal and vital NETosis can be triggered in COVID-19 [25]. Suicidal NETosis causes programmed cell death and the rupturing of the plasma membrane, which leads to the release of NETs in the extracellular space. Under stress, such as SARS-CoV-2 infection, the NADPH oxidase complex is activated for generating ROS. Following this, protein complexes known as azurosomes might experience dissociation in the case of ROS, which allows particles to be released by NE and myeloperoxidase (MPO) in the cytosol [26]. After the nuclear transport of MPO and NE, they decompose histones and laminin, causing condensation, destruction of the nuclear shell, and chromatin depolymerization. Subsequently, peptidyl arginine deiminase 4 (PADI4) causes the citrullination of histones and mediates chromatin decondensation. After decondensation, the deoxyribonucleic acid is transported in the cytoplasm to bind to granular proteins, extracellular histone H3 and cytoplasmic proteins for generating NET that is finally released outside the cells [27]. During vital NETosis, platelet activation results in SARS-CoV-2-mediated NET generation, and it promotes this process by interacting with neutrophils via Toll-like receptor 4 (TLR4) and extracellular vesicle-dependent processes. The bacterial cell wall component lipopolysaccharide (LPS) activates platelets and neutrophils via TLR4 [25]. Due to the abundance of NETs in alveolar capillaries, NET histones and cell-free DNA (cfDNA) accelerate the production of pro-inflammatory factors and additional comprehensive factors. After the excess release of NETs during COVID-19, alveolar microcirculation diseases can damage the lung tissue, causing severe disorders such as ARDS [28]. The effects of ROS on NETosis resulting from SARS-CoV-2 are shown in Fig. 1.

### Ferroptosis

Ferroptosis was proposed by Stockwell in 2012. It is a new type of PCD, mainly resulting from ROS-mediated lipid peroxidation [29]. Its main morphological features are mitochondrial abnormalities, including swelling and condensation, higher membrane density, low or no cristae, and outer membrane rupture [30, 31]. Ferroptosis might occur via two main pathways, including the external or transporter-mediated pathway (such as reduced cystine or glutamine absorption and elevated iron absorption) and the internal or enzyme-mediated pathway (such as inhibiting GPX4) [32]. Iron absorption can be triggered by transferrin receptor 1 (TfR1)-mediated endocytosis, where Fe^2+^ can be released in the cytoplasm through the divalent metal transporter 1 (DMT1) and six-transmembrane epithelial antigen of prostate 3 (Steap3). Intracellular iron can be preserved in ferritin (Fe^2+^ can be released through ferritinophagy) and delivered outside the cells via ferroportin. Iron overload can induce mitochondrial ROS production, thus damaging the cell membrane. Polyunsaturated fatty acids (PUFAs) can be converted into phospholipid hydroperoxides (PLOOHs) via a process known as lipid peroxidation that induces ferroptosis. This process is mediated by lipoxygenases (LOXs), lysophosphatidylcholine acyltransferase 3 (LPCAT3) and acyl-CoA synthetase long-chain family member 4 (ACSL4). The antioxidant system in cells can suppress ferroptosis, specifically the cystine glutathione (GSH)-glutathione peroxidase 4 (GPX4) axis can strongly inhibit lipid peroxidation. Several studies have shown that ferroptosis represents controlled cell death and is characterized by an increase in free iron levels related to ROS [33].

Ferroptosis is related to the pathogenic mechanism of COVID-19. Bednash et al. showed that ferroptosis occurs in the human heart and hamster lung tissues infected with SARS-CoV-2 [34]. Alterations in iron metabolic markers in blood samples (such as elevated ferritin and reduced serum iron levels) indicate iron overload and are related to severe COVID-19 [35]. Kronstein-Wiedemann et al. showed that SARS-CoV-2 can attack and destroy haemoglobin, which can cause iron release from porphyrins and discharge in circulation, accompanied by iron overload. To compensate for an increase in iron levels, a high level of ferritin is produced. As a result, the serum ferritin level may lead to hepatic cell death, which in turn can trigger iron release from ferritin, leading to an increase in systemic free iron levels. An increase in free iron levels can aggravate inflammation via ferroptosis and ROS-mediated oxidative damage [36]. Some COVID-19 manifestations, including hyperferritinemia, hypercoagulation, inflammation, and immune dysfunction, are also related to iron overload. Irrespective of the important role of iron in living cells, the iron overload-induced and dysregulation-induced free unbound iron shows extremely high reactivity and potential toxicity because of the effect on ROS production. These findings showed the crucial effect of ferroptosis on the COVID-19 pathogenic mechanism.

**Fig. 2:**
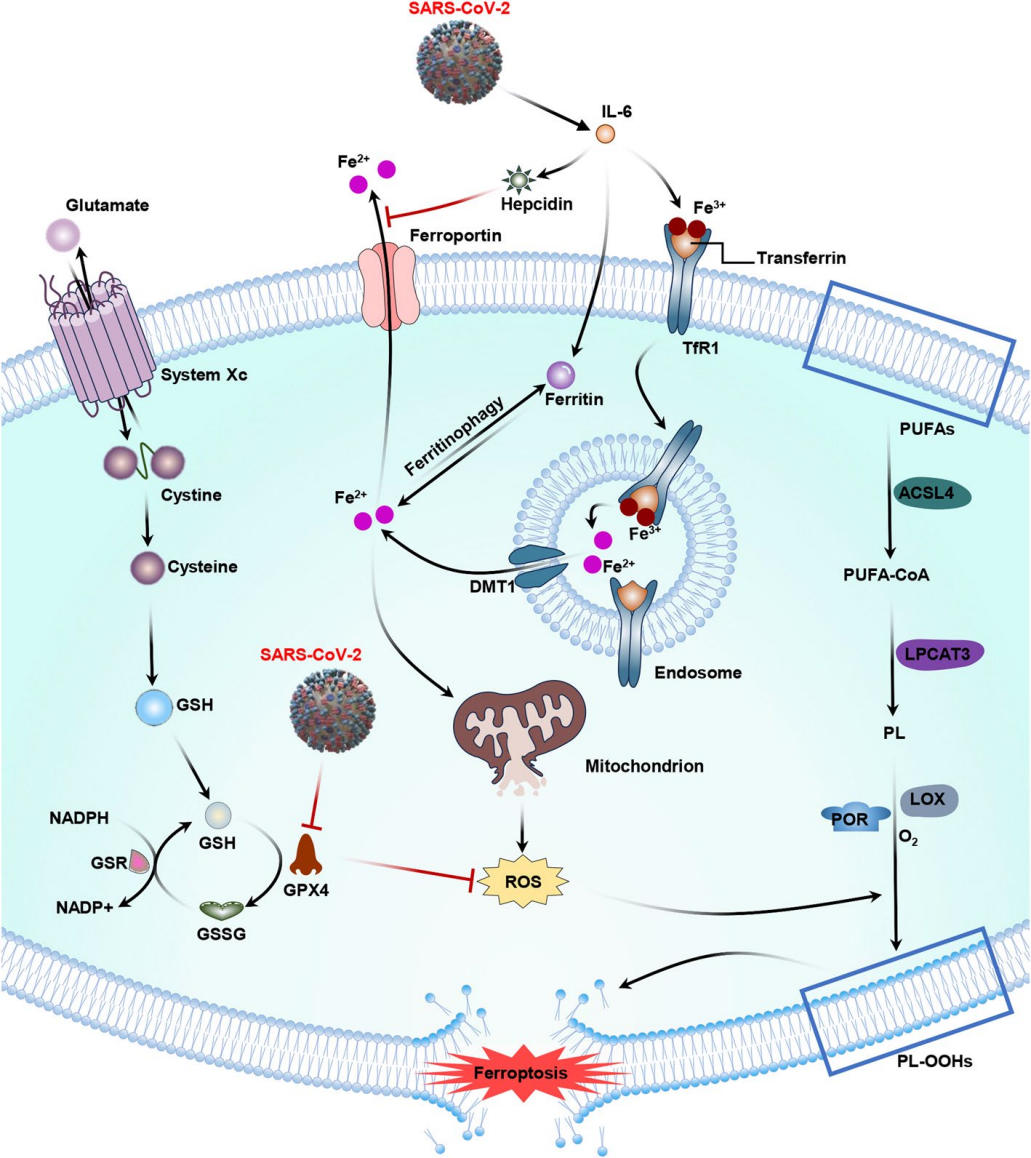
Effects of ROS on the ferroptosis pathway in SARS-CoV-2 infection. Ferroptosis results from lipid peroxidation caused by the imbalance between iron metabolism, antioxidant system, and ROS production. The IL-6 content increases in COVID-19 and promotes the increase in protein levels (hepcidin and transferrin). It also inhibits the exported protein ferroportin through hepcidin, causing ferroptosis. SARS-CoV-2 infection also negatively affects GPX4, thus triggering ferroptosis and lipid peroxidation. Activation effects are indicated by black arrows and inhibitory effects are indicated by red arrows

Furthermore, Li et al. reported that an increase in IL-6 level in the context of COVID-19 can directly promote ferritin expression and transferrin uptake, and suppress ferroportin export through hepcidin, resulting in ferroptosis [1]. A higher hepcidin level in serum was found to be related to the severity of COVID-19 [37]. SARS-CoV-2 can also decrease the expression of the GPX4 mRNA in infected Vero E6 cells [38]. Additionally, GSH expression decreases in patients with severe COVID-19. Due to GPX4 deficiency, GSH cannot be peroxidized to reduce lipid ROS generated through the Fenton reaction [39]. Consequently, ferroptosis and lipid peroxidation are induced through the accumulation of lipid ROS. The effects of ROS on SARS-CoV-2-induced ferroptosis are shown in Fig. 2.

### Apoptosis

Apoptosis is a main mode of PCD, morphologically characterized by nuclear condensation, cellular shrinkage, cytosolic membrane blebbing, chromosomal DNA fragmentation and the production of apoptotic bodies. Apoptosis plays an important role in several processes, such as embryogenesis and cellular homeostasis [40]. Apoptosis can result from extrinsic or intrinsic pathways (death receptor or mitochondrial pathway), which involves the cleavage (activation) of cysteinyl aspartate proteases (caspases). The death-inducing signaling complex (DISC) is generated after extracellular death ligands, such as Fas ligand (FasL), TNF-related apoptosis-inducing ligand (TRAIL) and tumour necrosis factor-alpha (TNF-α), bind to cell membrane death receptors and recruit the adaptor protein TNF receptor 1-associated death domain protein (TRADD) or Fas-associated protein with death domain (FADD), and the pro-cysteine-aspartic protease (procaspase)-8/10, which can lead to the activation of caspase-8 [41]. The activated caspase-8 exerts direct effects on cleaving procaspase-3/6/7 for inducing cell apoptosis. The activated caspase-8-processed BH3-interacting domain death agonist (Bid), in its truncated form, translocates into the mitochondria and leads to mitochondrial outer membrane permeabilization (MOMP) mediated by B-cell lymphoma-2 (Bcl-2)-associated X (BAX)/Bcl-2 homologous killer (BAK) to release apoptotic factors cytochrome c (Cytc) in the cytoplasm. Next, Cytc can combine with caspase-9 and apoptotic protease activating factor-1 (Apaf-1) to generate caspase-activating platform apoptosome, which can activate executioner caspase-3/7, resulting in apoptosis [42].

ROS activate intrinsic and extrinsic pathways. Pizzimenti et al. reported that reactive intermediates generated in the presence of oxidative stress led to the oxidation of PUFAs in the membrane lipid bilayers, resulting in aldehyde production [43]. Such lipid peroxidation products can interact with transcription factors, membrane receptors, or inhibitors to activate the intrinsic and extrinsic apoptotic pathways [44]. ROS can break the mitochondrial membrane by oxidizing PUFAs at an organelle level, leading to the swelling of the mitochondrial membrane, irreversible opening of the mitochondrial permeability transition pore and collapse of the mitochondrial membrane permeability. An increase in mitochondrial outer membrane permeability and mitochondrial-to-cytoplasmic release of Cytc can activate caspase-3 and induce cell apoptosis [45].

The level of ROS increases abnormally in virus-infected cells, which in turn might induce cell apoptosis [46]. For example, in HL-CZ cells, when apoptosis is caused by a Japanese encephalitis virus (JEV) infection, GRP78, p38 and p-ERK are activated, and ROS is generated, indicating that JEV plays a role in inducing apoptosis by the ERK/p38-ROS pathway [47]. Miao showed that the Hemagglutinating virus of Japan-envelope (HVJ-E) modulated the JNK, p38 and PI3K/beclin-1 pathways in prostatic cancer cells in a ROS-dependent manner, thus regulating apoptosis and autophagy [48]. Moreover, infection with porcine epidemic diarrhoea virus (PEDV) can induce time-dependent ROS accumulation and p53 activation in Vero cells. The activation of p53 and ROS accumulation are related to PEDV-mediated apoptosis, whereas p53 is modulated by ROS in a PEDV infection [49].

**Fig. 3:**
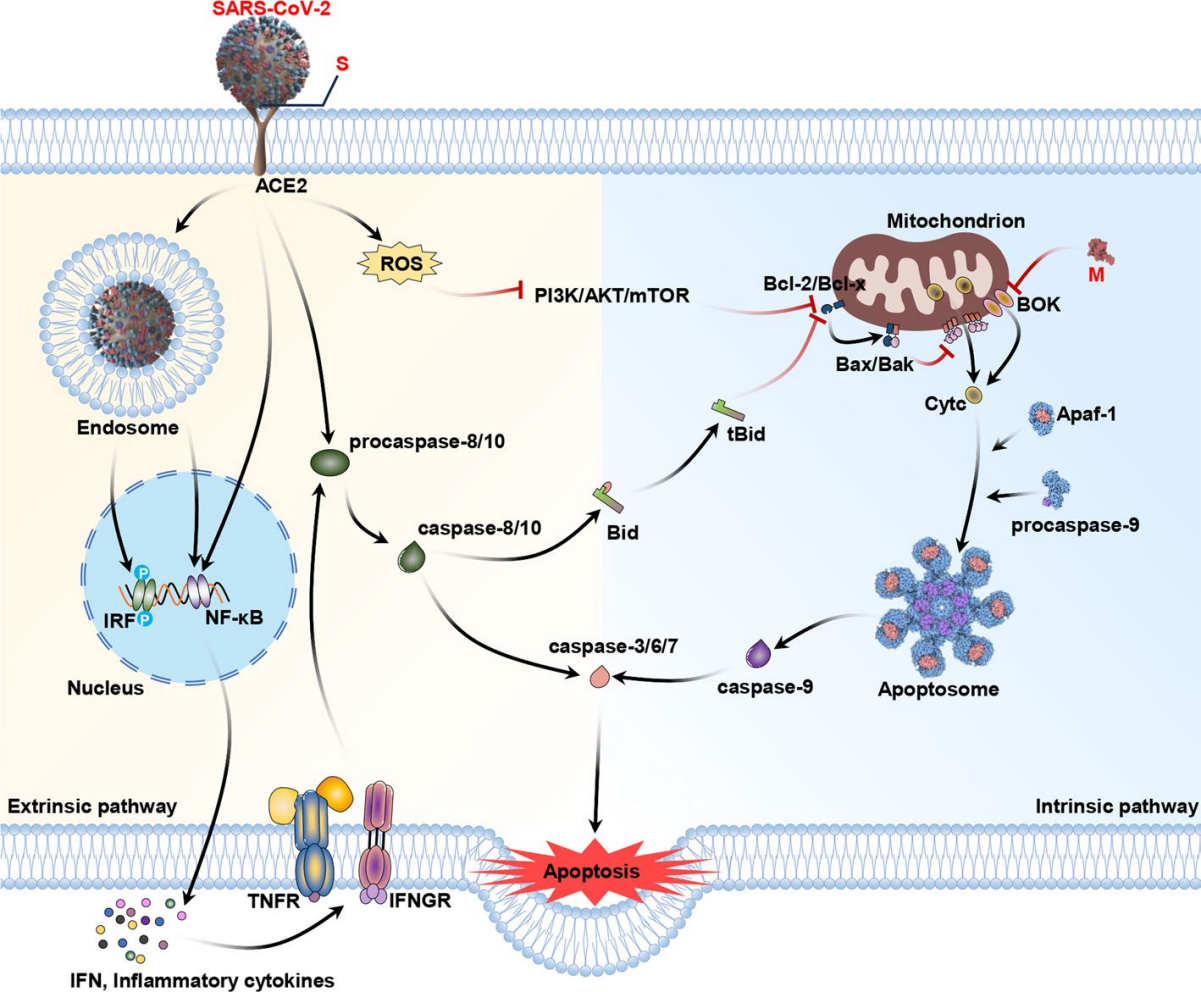
Effects of ROS on the apoptosis pathway in SARS-CoV-2 infection. SARS-CoV-2 can induce host cell apoptosis through intrinsic or extrinsic pathways. SARS-CoV-2 spike (S) upregulates intracellular ROS, later suppressing the PI3K/AKT/mTOR axis and inducing inflammation and apoptosis of infected cells. The SARS-CoV-2 S protein also modulates Bax and Bcl-2 for triggering the intrinsic apoptotic pathway, whereas, the M protein is responsible for stabilizing BOK for triggering intrinsic apoptosis. The activated death receptors contribute to recruiting and cleaving caspase-8/10, later leading to caspase-3/6/7 activation for inducing extrinsic cell apoptosis. Activation effects are indicated by black arrows, and inhibitory effects are indicated by red arrows

Several studies have shown that SARS-CoV-2 infection may cause apoptosis through various pathways. Additionally, apoptosis may be related to the COVID-19 pathogenic mechanism, which is associated with excess tissue and cell injury in the kidneys, liver, lungs, pancreas, immune system and nervous system. Li et al. reported that SARS-CoV-2 spike (S) can mediate autophagy by increasing intracellular ROS and later suppressing the PI3K/AKT/mTOR axis, causing apoptosis of infected cells and inflammatory response in such cells, highlighting that apoptosis performs pathogenic regulation during SARS-CoV-2 infection [50]. The SARS-CoV-2 M protein can also directly inhibit the ubiquitination of Bcl-2 ovarian killer (BOK) and enhance its mitochondrial translocation, resulting in the apoptosis of H292 cells by the intrinsic pathway [51]. ROS strongly affects cell signaling and regulates mitochondria-mediated major apoptosis pathways. However, the mechanism by which ROS regulates SARS-CoV-2 M-induced cell apoptosis needs to be elucidated. The roles of ROS in apoptosis due to SARS-CoV-2 infection are presented in Fig. 3.

### Pyroptosis

Pyroptosis is an inflammatory and lytic mode of PCD, which is characterized by cell swelling, balloon formation on the plasma membrane, release of cell content, destruction of the plasma membrane and cell lysis [52]. The molecular features of pyroptosis include inflammasome activation and assembly, maturation and release of pro-inflammatory cytokines, and formation of membrane pores. Pyroptosis strongly affects immunity and inflammation, thus, it is related to the occurrence of different diseases [53]. This type of cell death is regulated by the gasdermin D (GSDMD) protein family, under the activation and cleavage of proteases or caspase protein. The N-terminal fragments of the GSDMD protein cause membrane pore formation, which ruptures the cell membrane and induces cell lysis [54]. Pyroptosis is classified as a non-classical or classical inflammasome pathway depending on whether caspase-1 activation is needed for pyroptosis.

The classical inflammasome pathway mostly consists of inflammasome activation and assembly, porin formation and the maturation and secretion of IL-1β and IL-18. Damage-associated molecular patterns (DAMPs) and pathogen-associated molecular patterns (PAMPs) (including cholesterol crystals, extracellular ATP, viral dsDNA and bacterial LPS) accelerate inflammasome activation and assembly. Inflammasomes activate pro-caspase-1 through self-cleavage. The activated caspase-1 cleaves the porin GSDMD to produce the mature N-GSDMD; it also cleaves pro-IL-1β and pro-IL-18 to generate the mature IL-1β and IL-1 [55]. Unlike the classical inflammasome pathway, the non-canonical inflammasome pathway can be activated without inflammasome activation or assembly. LPS can activate caspase-4/5 (murine) and caspase-11 (human). The activated caspase-4/5/11 can cleave GSDMD to generate the mature N-GSDMD. Next, N-GSDMD integrates into the cell membrane and forms membrane pores, thus mediating pyroptosis.

Inflammasome activation and cell pyroptosis have important effects on the COVID-19 pathogenic mechanism. The above-mentioned process can not only induce excess tissue and cell injuries but also produce excess DAMPs and inflammatory cytokines, a process called cytokine storm, thus promoting the occurrence of COVID-19. ACE2, a key host receptor of SARS-CoV-2, is expressed in several types of cells. It mainly functions to convert angiotensin II (Ang II) into Ang (1–7) for counteracting the role of Ang II [56]. As ACE2 is mostly occupied by SARS-CoV-2, the degradation of Ang II can be reduced, resulting in its accumulation. However, hyperactivation of the Ang II-angiotensin 1 receptor (AT1R) pathway can cause ROS production and mitochondrial impairment, leading to NLRP3 inflammasome-associated pyroptosis, as shown by studies on endothelial progenitor cells (EPCs) and hematopoietic stem cells (HSCs) that were damaged after SARS-CoV-2 infection [57].

**Fig. 4:**
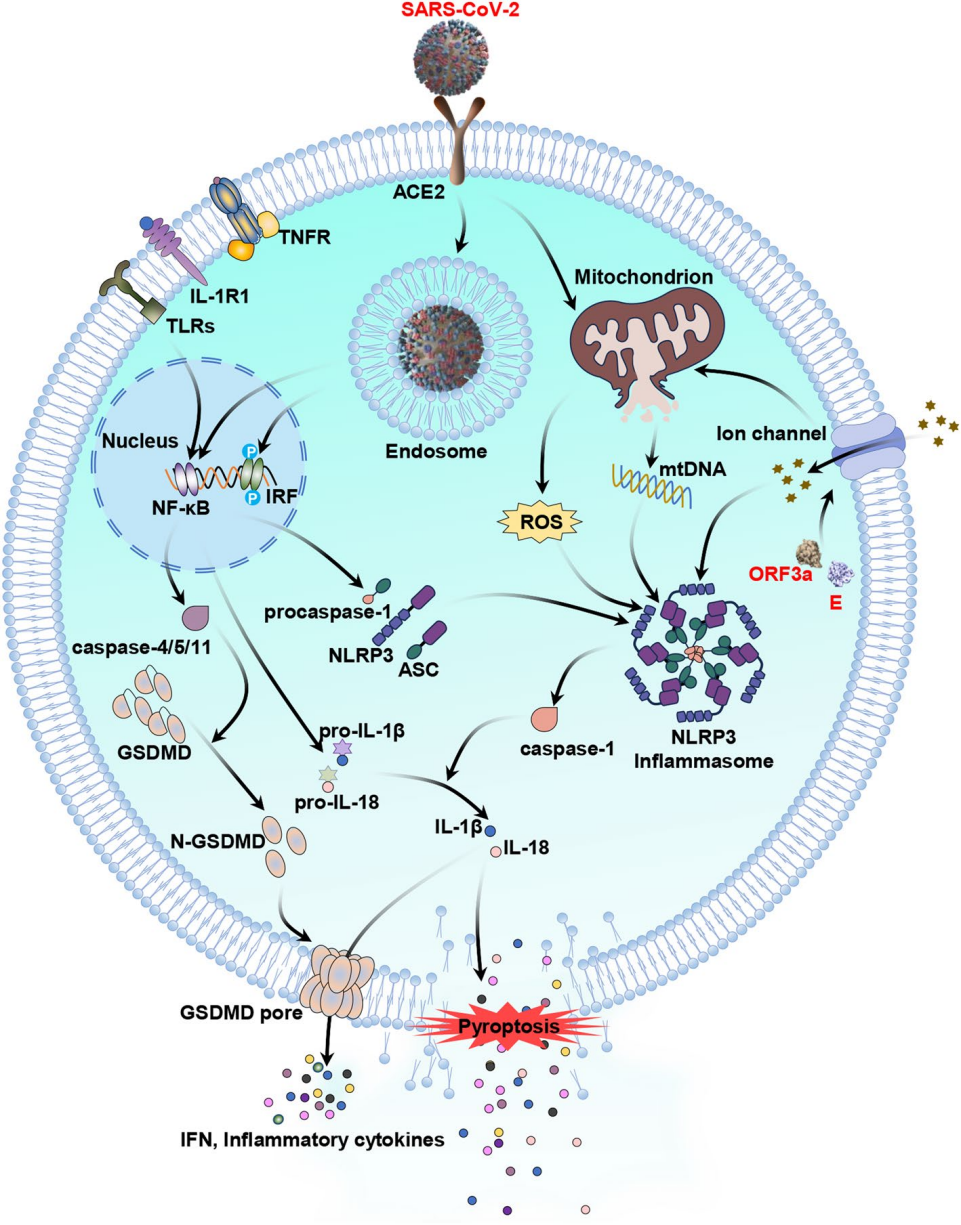
Effects of ROS on the pyroptosis pathway during SARS-CoV-2 infection. Activated TNFR, IL-1R1 and TLR pathways activates NF-κB to promote the production of pro-inflammatory factors (pro-IL-1β, pro-IL-18, NLRP3 and procaspase-1). DAMPs and PAMPs resulting from SARS-CoV-2 activates the NLRP3 inflammasome for cleaving procaspase-1, which can cleave pro-IL-1β and pro-IL-18. Caspase-1-cleaved GSDMD forms pores by inserting into the membrane, causing cell pyroptosis and the release of cellular contents. SARS-CoV-2-induced ROS generation and mtDNA release also leads to the activation of the NLRP3 inflammasome. Activation effects are indicated by black arrows

The activation of the inflammasome is a rate-limiting step in cell pyroptosis, and it is affected by various factors, including bacteria, viruses, toxins, ROS production, ATP alteration, ion imbalance, mitochondrial dysfunction, lysosomal destruction, etc. [58]. Studies have shown that different SARS-CoV-2-encoded proteins can modulate inflammasome activity via several mechanisms. For example, Xu et al. identified the SARS-CoV-2 E protein and ORF3a as viroporins associated with ion disease. SARS-CoV-2 E protein can increase Ca^2+^ influx, while ORF3a can trigger Ca^2+^ influx and K^+^ efflux, leading to an increase in Ca^2+^ level and a decrease in K^+^ level in cells [59]. Ion imbalance directly activates the NLRP3 inflammasome and promotes mitochondrial ROS production. Additionally, the absent of melanoma 2 (AIM2) inflammasome contributes to caspase-1 activation and pyroptosis, as found in human monocytes infected with SARS-CoV-2 [60]. Furthermore, Zheng et al. identified that the SARS-CoV-2 E protein activates the NLRP3-dependent inflammasome and TLR2 pathways, leading to the induction of pro-inflammatory factor generation through the activation of the NF-κB pathway [61]. During a SARS-COV-2 infection, mitochondrial DNA (mtDNA) and oxidized mtDNA (resulting from excessive ROS production) may be released by excess dysfunctional mitochondria, whereas cell-free DNA (cfDNA) may be released by dead cells. Cytoplasmic double-stranded DNA, including mtDNA, oxidized mtDNA and cfDNA, can directly trigger AIM2 inflammasome-induced pyroptosis [62]. The effects of ROS on pyroptosis following a SARS-CoV-2 infection are presented in Fig. 4.

### Autophagy

Autophagy (also known as type II PCD) was discovered by Ohsumi in yeast in 1992. In this process, proteins and organelles are degraded and recycled for maintaining intracellular homeostasis, and intracellular pathogens are eliminated [63]. Its primary morphological feature is the sequestration of cytoplasmic vacuoles and cargo in the double-membrane cytosolic vesicle, known as an autophagosome. Autophagy can be of three types, including macroautophagy (herein called autophagy), microautophagy and chaperone-mediated autophagy (CMA). In canonical autophagy, unwanted components are engulfed by subcellular membranes (including mitochondria, Golgi complex, endoplasmic reticulum [ER] and endosomes) to form a double-membrane vesicle called phagophore, which fully expands and surrounds its cargo before fusing with lysosomes to form the double-membrane autophagosome to be degraded in the acidic environment [64]. Autophagy is initiated and regulated by numerous autophagy-related gene (ATG)-encoded proteins, which mostly include (1) the Atg1/unc-51-like kinase (ULK) complex; (2) two ubiquitin-like protein (Atg8/LC3, Atg12) conjugation systems; (3) the class III phosphatidylinositol 3-kinase (PtdIns3K)/Vps34 complex I; and (4) two transmembrane proteins, i.e., VMP1 and Atg9/mAtg9 (and the relevant proteins like Atg18/WIPI-1 that are related to migration). Besides, the unwanted components can be labelled by the ubiquitin-binding protein sequestosome 1, p62 (SQSTM1) [65].

Autophagy is a self-digestion process that is responsible for degrading intracellular structures under stress, leading to cell survival. However, prolonged autophagy may cause cell death. Some studies have suggested that ROS is required for autophagy-induced cell death. For example, macrophages exposed to zVAD (a pan-caspase inhibitor) and treated with LPS caused activation of poly(ADP-ribose) polymerases (PARPs) after ROS accumulation, resulting in cell autophagy [66]. Rotenone (the mitochondrial complex I inhibitor) and TTFA (the complex II inhibitor) caused autophagy regulated through ROS generation in HEK 293, HeLa and U87 cells but not in non-transformed cells [67].

**Fig. 5:**
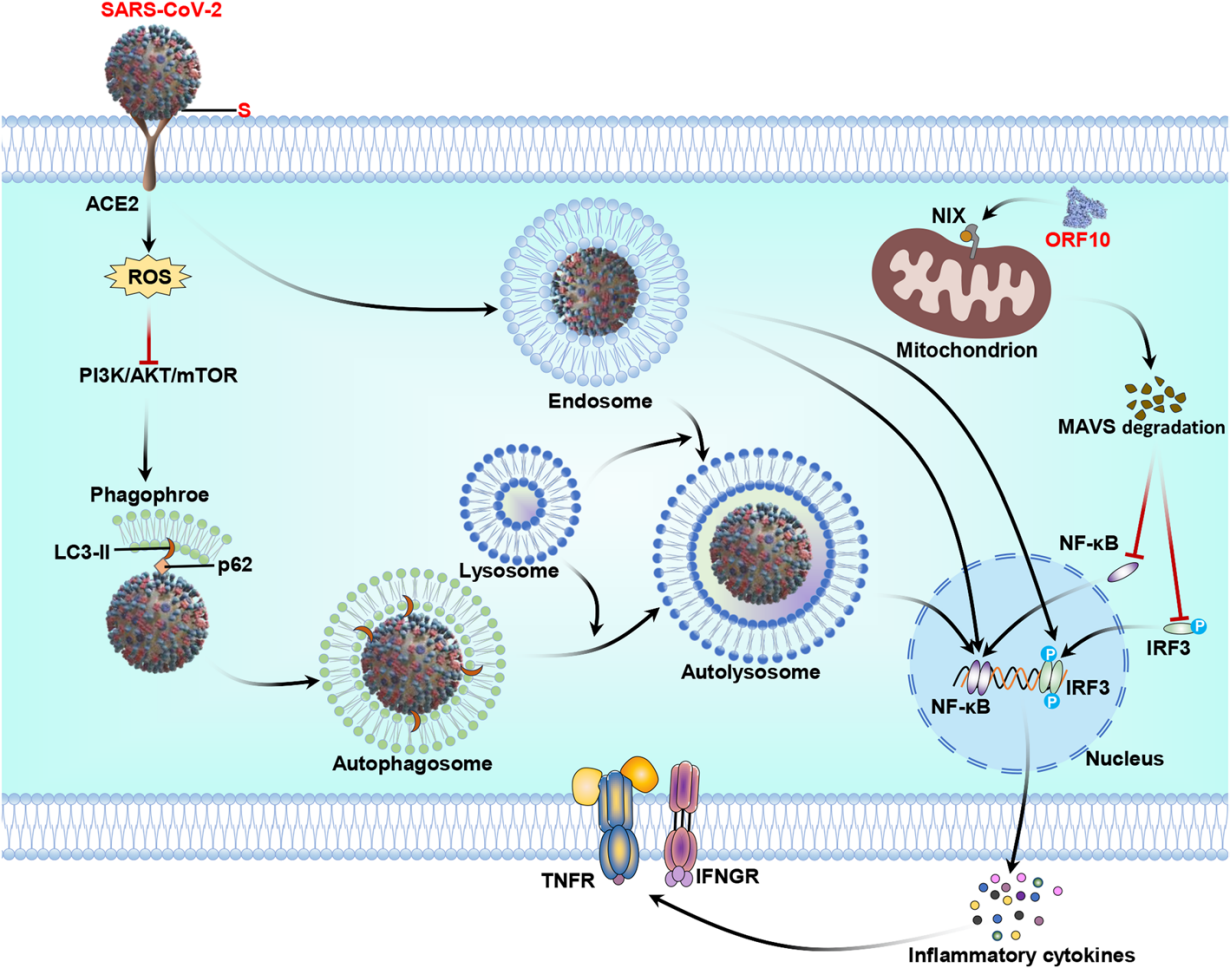
Effects of ROS on the autophagy pathway during SARS-CoV-2 infection. Subcellular membrane-derived phagophores expand and elongate to form an autophagosome, which can later fuse with lysosomes to form the autophagolysosome for degradation under the regulation of ATGs. The S protein of SARS-CoV-2 induces autophagy by increasing intracellular ROS levels and later suppressing the PI3K/AKT/mTOR axis, resulting in an inflammatory response. SARS-CoV-2 ORF10 directly interacts with the mitochondrial receptor Bcl-2 interacting protein 3 like (BNIP3L)/NIX to promote mitophagy. Mitophagy causes the degradation of mitochondrial antiviral signaling protein (MAVS), which regulates the activation of NF-kappaB (NF-κB) and IRF3 to upregulate immune response and inflammation-related genes in response to viral infection. Activation effects are indicated by black arrows, and inhibitory effects are indicated by red arrows

As of current evidence, it is shown that autophagy is a critical and intricate event that occurs during a SARS-CoV-2 infection. The mTOR pathway plays an important role in regulating cell autophagy. In a study, the proteotranscriptomic data showed that the phosphoinositide 3 kinase (PI3K)/AKT/mTOR pathway was activated following SARS-CoV-2 infection [68]. Li et al. reported that the S protein in SARS-CoV-2 can cause autophagy by increasing intracellular ROS levels and later suppressing the PI3K/AKT/mTOR axis, resulting in an inflammatory response and apoptosis of those infected cells. These findings suggested that SARS-CoV-2 S can increase ROS levels to induce cell injury. Autophagy can reduce ROS levels to mitigate oxidative damage [69]. Further studies are needed to determine if autophagy activation can revert the increased ROS level during SARS-CoV-2 infection. Additionally, the SARS-CoV-2 accessory protein open reading frame (ORF)10 directly interacts with the mitochondrial receptor Bcl-2 interacting protein 3-like (BNIP3L)/NIX (including LIR) to promote mitophagy [70]. The activation of mitophagy leads to the degradation of mitochondrial antiviral signaling protein (MAVS) [71], which upregulates the immune response and inflammation-related genes in response to viral infection by regulating the activation of NF-kappaB (NF-κB) and IRF3 [72]. Mitophagy decreases the production of ROS and mtDNA, thereby suppressing cytokine release and cell pyroptosis mediated by the NLRP3 inflammasome [73]. The effects of ROS on autophagy resulting from SARS-CoV-2 are presented in Fig. 5.

### Necroptosis

Necroptosis, a caspase-independent form of programmed necrotic cell death, can be initiated by various factors, including death receptor ligands tumour necrosis factor (TNF), Toll-like receptors (TLRs), interferons (IFNs), and viral infections [74]. Among the diverse triggers for necroptosis, TNF is the most extensively studied extracellular death ligand. Upon binding of TNF to its receptor TNFR1, a cascade of protein recruitment occurs, involving TRADD, FADD, receptor-interacting protein kinase 1 (RIPK1), RIPK3 and caspase-8, which collectively facilitate the assembly of the necrosome. In TNF-stimulated cells, the inactivation of caspase-8 results in the autophosphorylation of RIPK1 and RIPK3, which subsequently phosphorylates and promotes the oligomerization of MLKL. The oligomerized pMLKL forms pores in the cytoplasmic membrane, culminating in necroptosis [14]. Morphologically, necroptosis resembles necrosis, characterized by the clustering of dying cells, disrupted membranes, swollen cell bodies and organelles, and fragmented chromatin [75].

Accumulating evidence indicates that necroptosis plays a role in the pathogenesis of COVID-19. A recent study identified the activation of RIPK1 in human lung samples from patients with COVID-19. Xu et al. demonstrated that SARS-CoV-2 NSP12 directly interacts with RIPK1, facilitating its activation and subsequently inducing the transcription of proinflammatory factors and host factors, such as ACE2, thereby promoting viral entry into cells [76]. Recent reports indicate that serum RIPK3 levels are elevated in patients with severe COVID-19 compared with those with milder ones, implying a potential role for RIPK3 in the progression from COVID-19 pneumonia to ARDS [77]. Furthermore, research by Li et al. has demonstrated that SARS-CoV-2 can induce RIPK3-mediated cell necroptosis, which is significantly mitigated by RIPK3 inhibitors [78]. Collectively, these findings underscore the critical involvement of necroptosis in the pathogenesis of COVID-19.

ROS have long been recognized as pivotal mediators of necroptosis [79]. For instance, it has been shown that TNF can induce mitochondrial ROS, which in turn facilitate necrosome formation [80]. Either the elimination of ROS by scavengers such as butylated hydroxyanisole or the inhibition of the electron transport chain by inhibitors such as amytal can suppress TNF-induced necroptosis [81, 82]. As one of the critical cell death pathways in SARS-CoV-2 infection, necroptosis can be harnessed to mitigate cytokine storm damage [83]. Identifying drugs that specifically inhibit COVID-19-related necrosis may significantly enhance therapeutic strategies for this disease. Necrostatin-1 is a specific RIPK1 inhibitor that suppresses the necroptosis signaling pathway. Recent studies suggest that necrostatin-1 may play a role in mitigating complications related to COVID-19 by suppressing necroptosis, reducing DAMP and pro-inflammatory cytokine release, inhibiting the inflammatory NF-κB pathway and decreasing ROS damage [84].

**Fig. 6:**
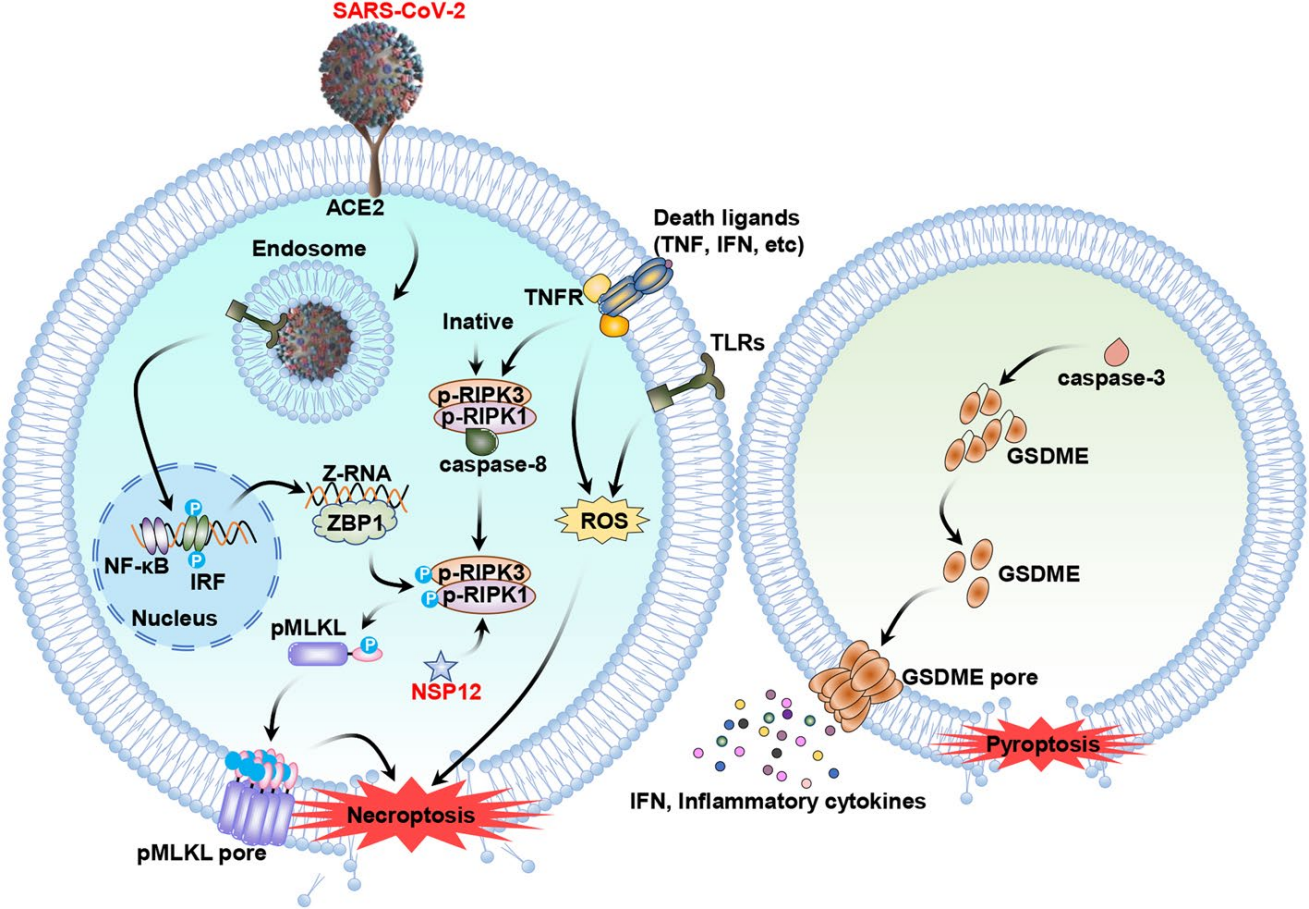
Effects of ROS on the necroptosis pathway during SARS-CoV-2 infection. SARS-CoV-2 infects host cells via the ACE2 receptor, leading to the production of Z-RNA within the infected cells (indicated in blue). The virus-derived Z-RNA is detected by the host protein ZBP1, which subsequently initiates necroptosis and promotes the phosphorylation and translocation of MLKL. In bystander cells that are not infected by SARS-CoV-2 (indicated in green), caspase-3 is activated, resulting in the cleavage of GSDME. The cleaved GSDME then forms pores in the plasma membrane. Additionally, SARS-CoV-2 NSP12 can interact directly with and stimulate RIPK1 activation, thereby triggering necroptosis. Activation effects are indicated by black arrows

The consequence of necrosis is the release of various cellular components, including nucleic acids, ATP and ROS, which may subsequently activate other receptors expressed in bystander cells, such as inflammasome-associated sensors. Liang et al. demonstrated that necroptosis is the primary cell death in cells infected with SARS-CoV-2, while uninfected bystander cells primarily undergo apoptosis, with pyroptosis occurring at a later stage of infection. Mechanistically, SARS-CoV-2 targets lung epithelial cells via the ACE2 receptor and produces Z-RNA within the infected cells. The host protein ZBP1 detects virus-derived Z-RNA, subsequently triggering necroptosis as well as the phosphorylation and translocation of MLKL. In bystander cells not infected by SARS-CoV-2, caspase-3 activation leads to the cleavage of GSDME, resulting in the initiation of an alternative GSDME-mediated pyroptotic cell death pathway [85]. The emergence of novel SARS-CoV-2 variants of concern (VOC) poses a significant global threat due to their mutations, which impact transmissibility and facilitate immune evasion. This research group further elucidates that the Delta (B.1.617.2) variant, which results in more severe disease manifestations in humans compared with the Omicron (B1.1.529) variant, is correlated with markedly elevated levels of Z-RNA/ZBP1 interactions, necroptosis and disease severity in animal models [85]. Additionally, a recent study showed that, compared with the natural ORF3a Beta variant Q57H of SARS-CoV-2, the deletion of the residue G188 (∆G188) variant significantly yielded more robust production of ROS and pro-inflammatory responses, leading to enhanced apoptosis and necrosis [86]. The effects of ROS on necroptosis following a SARS-CoV-2 infection are presented in Fig. 6.

## Possible therapies related to ROS in COVID-19

As high ROS levels are closely related to viral infection, replication, inflammation and oxidative damage, modulating ROS levels in patients with COVID-19 might be effective in treating hyperinflammation, protecting tissues against oxidative damage and suppressing viral replication [87]. The use of ROS inhibitors in the treatment of viral infections, including HCV, influenza virus (IV) and human immunodeficiency virus (HIV), can reduce viral replication and pathogenesis in animal models in vivo and in vitro. Randomized controlled trials need to be performed to validate clinical results, especially of those studies that have shown that targeting ROS-generating pathways might be an effective broad-spectrum antiviral strategy. Designing a treatment method for a disease with unknown pathogeny remains challenging. If oxidative stress is identified as an important step in the mechanism underlying SARS-CoV-2 infection, using antioxidants and drugs that inhibit ROS production, such as NET inhibitors, tocilizumab, iron chelators, glutathione, melatonin, etc., can assist in the recovery of patients suffering from COVID-19.

### NET inhibitors

Suppressing NETosis might be an effective way to treat COVID-19 [88]. The main strategy involves inhibition of the basic components related to NETosis, including PAD4, NE, GSDMD and MPO [25]. Sivelestat, an important NE inhibitor approved by Japan and Korea, can mitigate acute lung injury. Maki reported that sivelestat showed favourable effects on ARDS patients [89], suggesting that the NE inhibitor was beneficial to patients with COVID-19. Additionally, a randomized, placebo-controlled phase Ib/II clinical trial regarding the treatment of COVID-19 with ARDS using Alvelestat was conducted, but no result was published (NCT04539795). Dornase alpha was approved by the FAD as the recombinant human DNase I. It has strong antiviral effects and can mitigate the effect of SARS-CoV-2 infection in vivo and in vitro [90]. Some clinical trials reported an association between dornase alpha and COVID-19 (NCT04432987, NCT04402970, NCT04387786, NCT04359654, NCT04432987, NCT04387786, etc.). In another non-randomized phase III clinical trial (NCT04402970), inhalation of dornase alpha generated favourable results, including reduced NETs and increased oxygenation, among patients with COVID-19 [91].

The protein cAMP-specific PDE4 shows high expression in neutrophils and can aggravate their inflammation. Therefore, some PDE inhibitors were identified as suitable candidates for developing anti-COVID-19 therapeutic agents. Some phase III clinical trials are underway, in which the PDE4 selective-inhibitor apremilast (NCT02735707) and ensifentrine (NCT04590586) are being tested. Dipyridamole and pentoxifylline were approved by the FDA as non-specific PDE inhibitors. Dipyridamole can inhibit SARS-CoV-2 replication among infected cells, which facilitates disease mitigation and substantially decreases D-dimer among patients with COVID-19 [92]. Phase II (NCT04391179) and III (NCT04410328) randomized clinical trials were conducted to evaluate the effectiveness of dipyridamole in treating patients with COVID-19; however, no results were published. In patients with COVID-19 treated with pentoxifylline, the lymphocyte count increased and the serum lactate dehydrogenase (LDH) concentration decreased [93]. A clinical study (NCT04433988) to assess the effectiveness of pentoxifylline in treating COVID-19 is underway.

Agents against inflammation and oxidation, including hydroxychloroquine, exert negative effects on SARS-CoV-2 replication, partly by inhibiting the NF-κB pathway. Suppression of NF-κB can induce adverse reactions because of immunosuppression. Hydroxychloroquine can suppress NET generation by preventing TLR9 from binding to its ligand and suppressing IL-8 and ROS generation. Additionally, hydroxychloroquine also suppresses PAD4 expression [94]. Autophagy is closely associated with platelet-mediated NET generation; administering hydroxychloroquine, an autophagy inhibitor, in patients with COVID-19 can inhibit NETosis to suppress immunothrombosis (NCT04434144 and NCT04351620) [95].

### Tocilizumab

The cytokine IL-6 plays a key role in cytokine storm development and the pathogenic mechanism underlying COVID-19. An increase in IL-6 content is a risk factor for the severity of COVID-19 and mortality of affected patients [96]. Tocilizumab, a humanized monoclonal antibody against membrane-bound and soluble IL-6 receptors approved by the FDA, can suppress IL-6 binding. The soluble IL-6 receptor is used for treating giant cell arteritis (GCA), cytokine release syndrome, rheumatoid arthritis (RA), polyarticular juvenile idiopathic arthritis (pJIA) and systemic sclerosis-associated interstitial lung disease (SSc-ILD) [97]. A study conducted with 20 patients with RA who were administered subcutaneous tocilizumab (162 mg/week for 6 months) found that tocilizumab reduced leukocyte-induced oxidative stress and promoted endothelial functions. Tocilizumab also reduced the low-density granulocyte frequency while inhibiting NET generation. In a randomized, placebo-controlled trial, administering tocilizumab led to better outcomes among patients with severe COVID-19 in retrospective observational cohort studies and case reports (NCT04320615) [98]. A large number of clinical trials of tocilizumab in COVID-19 pneumonia have been conducted (NCT04346355, NCT04356937, NCT04372186, NCT02735707 and NCT04381936).

### Iron chelators

High levels of free iron may aggravate inflammation via ROS-mediated ferroptosis and oxidative damage. Untreated ferroptosis can enhance various responses, which can aggravate inflammation, cause pulmonary injury, decrease lung capacity and result in multiple organ failure. Free-iron may promote hypercoagulation detected among severe patients with COVID-19 by non-enzymatically converting fibrinogen into fibrin clots by generating hydroxyl radicals. People with blood group O were found to have a lower risk of COVID-19 compared with those with other blood groups, which might be associated with lower serum iron levels [99].

Cellular iron homoeostasis is important for the invasion and survival of viruses, as shown by the dependence of viral replication on iron and the effect of viruses on modulating host iron metabolism [100]. Further studies are needed to investigate the significance of iron-chelation treatments and its relationship with viral infection. One strategy involves the use of iron chelators to bind free irons or deplete iron from iron-containing proteins. Deferoxamine is an injected iron chelator approved by the FDA. It can bind to free iron or iron in lysosomal ferritin and generate a stable complex that can be eliminated via kidneys [101]. The trials of deferoxamine in treating COVID-19 are underway (NCT04333550, NCT04361032 and NCT04389801). Deferiprone is another iron chelator that has been approved by the FDA for oral administration, and it can perform iron chelation more effectively than deferoxamine in the heart. Clinical studies on iron chelation treatment against COVID-19 are under investigation (NCT04333550, NCT04361032 and NCT04389801). Lipid peroxidation is closely associated with ferroptosis; therefore, blockage of these key enzymes (including ACSL4 and LOX263) might be an effective anti-COVID-19 treatment strategy. Pioglitazone and troglitazone, approved by the FDA for treating diabetes, can decrease viral replication and cell ferroptosis by selectively inhibiting the activity of ACSL4, following a SARS-CoV-2 infection [102]. A phase IV randomized clinical trial is recruiting patients with COVID-19 complicated with type 2 diabetes to assess the effectiveness of pioglitazone (NCT04604223). A phase four randomized clinical trial was completed in which patients with COVID-19 with type 2 diabetes were administered pioglitazone; however, no results were published (NCT04535700). Another trial in which patients with COVID-19 were administered tocilizumab for iron-chelation is ongoing (NCT04361032).

### Glutathione (GSH)

Glutathione is a soluble antioxidant, which can remove H_2_O_2_ and ROS and activate important antioxidant enzymes, such as glutathione peroxidases, thioredoxins and peroxiredoxins. GSH deficiency can induce higher susceptibility to COVID-19 infection among the elderly and can promote the development of concurrent diseases such as hypertension, obesity and diabetes [103]. Additionally, GSH can mitigate SARS-CoV-2 infection, as well as its viral load, which inhibits oxidative stress, thrombosis and the production of pro-inflammatory factors (IL-6, TNF-α and IL-8); it can also boost the immune system [104]. Oral or intravenous GSH (2 g) was found to efficiently mitigate dyspnoea among patients with COVID-19 [105], indicating that GSH supplements may be useful when the GSH level is lower than normal. Further clinical trials using GSH alone or in combination for treating COVID-19 are underway (NCT04703036 and NCT05371288).

The GSH precursor *N*-acetylcysteine (NAC) has anti-ferroptotic effects via the direct reinforcement of the cystine–GSH–GPX4 axis and the reduction of IL-6-induced ROS production [106], which is related to its efficiency in treating COVID-19. NAC may also modulate inflammation and T-cell response [107]. Ibrahim et al. conducted a cohort study and reported that intravenous NAC induced clinical improvements in ten patients (38–71 years old) with severe COVID-19 who were being supported by mechanical ventilation. Among these patients, one suffered from glucose-6 phosphate dehydrogenase (G6PD) deficiency. Intravenous NAC considerably decreased different inflammatory markers, such as ferritin and CRP. Finally, eight of their enrolled patients were discharged, while the remaining two patients showed clinical improvements at the time of publication [108]. However, according to one randomized, double-blind, placebo-controlled trial, treatment with a high dose of NAC could not avoid ARDS progression in patients with COVID-19 [109]. Several clinical trials were conducted to evaluate the effect of NAC on patients with COVID-19; however, the results are pending (NCT04803227, NCT05371288, NCT04703036, NCT04374461, NCT05074121 and NCT04455243). Other clinical trials using NAC alone or in combination for treating COVID-19 are under investigation (NCT04928495 and NCT05736887).

### Vitamin C, vitamin E, and selenium

Vitamin C, or ascorbic acid, is a water-soluble vitamin that acts as an essential co-factor for dietary iron absorption, carnitine and catecholamine metabolism, and collagen biosynthesis. Some studies have shown that vitamin C deficiency can result in inflammation and oxidative stress, and can impair immunity. A phase II clinical trial was conducted to assess the effectiveness of vitamin C in patients with COVID-19 to reduce COVID-19-related mortality by decreasing excess activation of the inflammatory response (NCT04264533) [110]. In COVID-19 inpatients, vitamin C may not improve their primary composite outcome of hospital survival or organ support-free days (NCT04401150 and NCT02735707) [111]. Further clinical trials on vitamin C are ongoing (NCT05694975, NCT05029037, NCT04335084 and NCT04468139). Multiple mechanisms are related to the role of vitamin C in reducing the severity of COVID-19. For example, it can promote wound healing, endothelial integrity and lung endothelial barrier, thus decreasing ARDS or oxidative stress. Zuo et al. showed that vitamin C decreased the entry of the SARS-CoV-2 virus by reducing the number of ACE2 receptors on the endothelial cells [112]. It can also promote the generation of IFNs to enhance antiviral reactions and suppress the NF-κB signaling pathway [113]. Vitamin C can also reduce the formation of NETs, which is related to blood clotting and vascular damage. Of note, vitamin C can act as an antioxidant and effectively mitigate the phorbolmyristate acetate (PMA)-mediated NETosis in normal neutrophils by scavenging ROS [114]. Thus, vitamin C probably suppresses NET generation and NETosis in patients with COVID-19.

Selenium and vitamin E are strong antioxidants. A retrospective study showed that the selenium level is correlated with the COVID-19 recovery rate among Chinese patients [115]. Some observational and epidemiological studies reported that selenium and vitamin E deficiencies promote virus pathogenicity and reduce immune responses. Similarly, supplementing these two nutrients can enhance the resistance to respiratory infection [116]. The results showed that selenium levels were positively associated with clinical complications and outcomes [115], indicating the effectiveness of selenium supplementation in treating patients with COVID-19. Selenium can also upregulate GPX4 to protect cells against ferroptosis in the stroke model [117] and can be used for treating COVID-19 (NCT04869579, NCT04798677, NCT04751669 and NCT04323228). Administering selenium along with vitamin E in patients with COVID-19 can promote IL-2 production and enhance the CD4^+^ and CD8^+^ T-cell activities, thereby decreasing the risk of infection. They can suppress ROS generation through the corresponding antioxidant effects [100]. One case–control study was performed to determine the effectiveness of vitamin E in treating inpatients with COVID-19, but the results have not been published (NCT05946499). Selenium and vitamin E are important for enhancing the immune response and antioxidant activity to resist SARS-CoV-2 infection; however, more suitable clinical studies are needed to determine the efficacy.

### Melatonin

Melatonin is a strong MPO inhibitor with excellent activities, such as anti-inflammation, anti-oxidation and neuroprotection. Several studies have shown that ROS production, as well as, neutrophil and MPO activities have critical effects on the inflammatory immune response. The stimulation of NET formation depends on ROS production and MPO processing; thus, suppressing MPO might suppress the generation of NETs [118]. Melatonin serves as a key regulatory factor and reversible inhibitor of MPO activity and exerts a strong effect on ROS detoxification. Hence, inhibiting MPO and eliminating unnecessary ROS are the key strategies for treating patients with COVID-19. Melatonin was found to be a safe anti-COVID-19 therapeutic agent, and it is administered along with the FDA emergency-authorized cocktail, REGEN-COV2, for inhibiting COVID-19 progression [119]. The production of ROS and reactive nitrogen species (RNS) through the cytokine storm during COVID-19 may be related to the upregulation of matrix metalloproteinase (MMP) [120]. MMPs are enzymes dependent on calcium and zinc. They are related to the remodelling of the extracellular matrix, and they also promote cytokine and chemokine activation in leukocytes [121]. ROS production in activated macrophages can activate and upregulate MMPs. However, melatonin can decrease the protein and mRNA levels of MMPs and increase the levels of their inhibitor in tissues [122]. These findings indicate that melatonin is a promising candidate for treating patients with COVID-19. Further clinical trials on the exact role of melatonin in COVID-19 treatment are underway (NCT04470297 and NCT04409522).

## Conclusions and perspectives

At the beginning of the COVID-19 pandemic, ROS was found to strongly influence viral replication and inflammatory response. Antioxidants, with anti-inflammatory and antiviral activities, were found to be effective in treating the cytokine storm against COVID-19. Cell death is important for the mechanism underlying the pathogenesis and the maintenance of host homeostasis. Following the COVID-19 outbreak, researchers found that SARS-CoV-2 induced cell mortality by different modes of cell death and cell autophagy for their benefits [1, 14]. ROS plays a key role in the mechanisms underlying the pathogenesis of human diseases by accelerating cell death, such as pyroptosis, apoptosis, NETosis and ferroptosis, necroptosis and by blocking pro-survival signals, such as unfolded protein response and autophagy. Cells may undergo various forms of cell death depending on the specific stimulus encountered. Mechanistically, apoptosis is initiated by the activation of initiator caspases, such as caspase-8/9/10, which subsequently activate executioner caspases, including caspase-3 and caspase-7, to drive the cell death process [42]. Pyroptosis is executed by pore formation mediated by members of the gasdermin family, which can be triggered through inflammasome activation and subsequent caspase-1 cleavage of GSDMD [123]. Necroptosis is characterized by RIPK3-mediated oligomerization of MLKL, leading to the formation of MLKL pores in the cell membrane, thereby executing cell death [75]. Further evaluation of the above mechanisms can facilitate precise real-time prognosis and help identify new interventional targets. Information on these parameters can help manage the COVID-19 pandemic and lay the foundation for the effective treatment of other prominent infections.

Several vaccines have been developed to prevent the spread of SARS-CoV-2, but therapeutic agents against SARS-CoV-2 infections are mostly conventional antiviral agents. Thus, the pathogenesis related to SARS-CoV-2 infection needs to be evaluated to develop effective agents for treating COVID-19 [50]. As ROS strongly affect COVID-19 development by promoting cell death, several potential drugs and treatments were proposed and tested in clinical studies (Table 1). Oxidative stress induced by ROS is associated with major alterations that occur in some infectious and inflammatory disorders, which are the common factors that occur in all the above-mentioned events. We believe that such an understanding may not only prove of significant benefit to quell the present COVID-19 pandemic, but also provide a basis for more efficient treatment of other significant infections going forward.

**Table 1:** Therapeutic targets and drugs associated with ROS in cell death for SARS-CoV-2

Drug name	Category	Targets	Effect on cell death	Mechanism	Clinical trial ID
Sivelestat	NET inhibitor	NE	NETosis	A selective NE inhibitor [89]	Retrospective cohort study
Alvelestat	NET inhibitor	NE	NETosis	Inhibits NE	NCT04539795
Dornase alpha	NET inhibitor	DNA	NETosis	Catalyses the degradation of extracellular DNA [90, 91]	NCT04432987 NCT04402970 NCT04387786 NCT04359654 NCT04432987 NCT04387786 NCT04402970
Apremilast	PDE inhibitor	PDE4	NETosis	A PDE4 selective inhibitor	NCT02735707
Ensifentrine	PDE inhibitor	PDE4	NETosis	A PDE4-selective inhibitor	NCT04590586
Dipyridamole	Pan PDE inhibitor	PDE	NETosis	A PDE inhibitor [92]	NCT04391179 NCT04410328
Pentoxifylline	Pan PDE inhibitor	PDE	NETosis	A non-specific PDE inhibitor [92]	NCT04433988
Hydroxychlor- oquine	Antioxidant	NF-κB	NETosis	Inhibits the NF-κB pathway	NCT04434144 NCT04351620
	PAD4 inhibitor	PAD4		Inhibits NET formation[94, 95]	
Tocilizumab	IL-6 receptor antagonist	IL-6 receptor	NETosis	Antagonizes both soluble and membrane-bound IL-6 receptors to prevent IL-6 binding	NCT04320615 NCT04346355 NCT04356937 NCT04372186 NCT02735707 NCT04381936 NCT04361032
	Iron chelator	Iron ion	Ferroptosis	Iron chelation [99]	
Deferoxamine	Iron chelator	Iron ion	Ferroptosis	Binds with free iron and iron from lysosomal ferritin [101]	NCT04333550 NCT04361032 NCT04389801
Deferiprone	Iron chelator	Iron ion	Ferroptosis	Binds with free iron	NCT04333550 NCT04361032 NCT04389801
Pioglitazone	Antidiabetic agent	ACSL4	Ferroptosis	Inhibits ACSL4 activity	NCT04604223 NCT04535700
Troglitazone	Antidiabetic agent	ACSL4	Ferroptosis	Inhibits ACSL4 activity [102]	/
GSH	Antioxidant	ROS	Ferroptosis	Neutralizes harmful oxidative stress [104]	NCT04703036 NCT05371288
NAC	Precursor of GSH	GSH-GPX4	Ferroptosis	Reinforces the cystine–GSH–GPX4 axis	NCT04803227 NCT05371288 NCT04703036 NCT04374461 NCT05074121 NCT04455243 NCT04928495 NCT05736887
		ROS		Reduces the IL-6-promoted ROS production [106]	
Vitamin C	Antioxidant	ACE2	NETosis	Downregulates the amount of ACE2 receptor [111–114]	NCT04264533
		ROS		Increases production of interferons; scavenges ROS	NCT04401150
					NCT02735707
					NCT05694975
					NCT05029037
					NCT04335084
					NCT04468139
Selenium	Antioxidant	GPX4	Ferroptosis	Induces GPX4 expression	NCT04869579
		ROS		Inhibits ROS production [115–117]	NCT04798677
					NCT04751669
					NCT04323228
Vitamin E	Antioxidant	ROS	Ferroptosis	Inhibits ROS production [100, 115, 116]	NCT05946499
Melatonin	MPO inhibitor	MPO	NETosis	Inhibits MPO and detoxifies ROS [122]	NCT04470297 NCT04409522
					NCT05946499
Vitamin C	Antioxidant	ACE2	NETosis	Downregulates the amount of ACE2 receptor [111–114]	NCT04264533
					NCT04401150
			ROS	Increases production of interferons; scavenges ROS	NCT02735707
					NCT05694975
					NCT05029037
					NCT04335084
					NCT04468139
Selenium	Antioxidant	GPX4	Ferroptosis	Induces GPX4 expression	NCT04869579
					NCT04798677
		ROS		Inhibits ROS production [115–117]	NCT04751669
					NCT04323228
Vitamin E	Antioxidant	ROS	Ferroptosis	Inhibits ROS production [100, 115, 116]	NCT05946499
Melatonin	MPO inhibitor	MPO	NETosis	Inhibits MPO and detoxifies ROS [122]	NCT04470297 NCT04409522

NET, extracellular traps; NE, neutrophil elastase; PDE4, phosphodiesterase 4; NF-κB, NF-kappaB; IL-6, interleukin 6; ACSL4, acyl-CoA synthetase long-chain family member 4; ROS, reactive oxygen species; GSH, glutathione; NAC, *N*-acetylcysteine; GPX4, glutathione peroxidase 4; ACE2, angiotensin-converting enzyme-2; MPO, myeloperoxidase; NADPH, nicotinamide-adenine dinucleotide phosphate; ND, ND not determined; RAS, renin-angiotensin system

## Data Availability

Not applicable.
